# The E3 Ubiquitin Ligase RNF5 Facilitates SARS-CoV-2 Membrane Protein-Mediated Virion Release

**DOI:** 10.1128/mbio.03168-21

**Published:** 2022-02-01

**Authors:** Zhen Yuan, Bing Hu, Hurong Xiao, Xuan Tan, Yan Li, Ke Tang, Yonghui Zhang, Kun Cai, Binbin Ding

**Affiliations:** a Department of Biochemistry and Molecular Biology, State Key Laboratory for Zoonotic Diseases, School of Basic Medicine, Tongji Medical College, Huazhong University of Science and Technology, Wuhan, Hubei, China; b Cell Architecture Research Institute, Huazhong University of Science and Technology, Wuhan, Hubei, China; c Institute of Health Inspection and Testing, Hubei Provincial Center for Disease Control and Prevention, Wuhan, Hubei, China; d Department of Pathogen Biology, School of Basic Medicine, Tongji Medical College, Huazhong University of Science and Technology, Wuhan, Hubei, China; e Hubei Key Laboratory of Natural Medicinal Chemistry and Resource Evaluation, School of Pharmacy, Tongji Medical College, Huazhong University of Science and Technology, Wuhan, Hubei, China; Columbia University/HHMI

**Keywords:** release, ubiquitination, RNF5, SARS-CoV-2, autophagy, virus-like particles

## Abstract

As an enveloped virus, severe acute respiratory syndrome coronavirus 2 (SARS-CoV-2) contains a membrane protein (M) that mediates viral release from cellular membranes. However, the molecular mechanisms of SARS-CoV-2 virion release remain poorly understood. In the present study, we performed RNA interference (RNAi) screening and identified the E3 ligase RNF5, which mediates the ubiquitination of SARS-CoV-2 M at residue K15 to enhance the interaction of the viral envelope protein (E) with M, whereas the deubiquitinating enzyme POH1 negatively regulates this process. The M-E complex ensures the uniform size of viral particles for viral maturation and mediates virion release. Moreover, M traffics from the Golgi apparatus to autophagosomes and uses autophagosomes for virion release, and this process is dependent on RNF5-mediated ubiquitin modification and M-E interaction. These results demonstrate that ubiquitin modification of SARS-CoV-2 M stabilizes the M-E complex and uses autophagosomes for virion release.

## INTRODUCTION

Severe acute respiratory syndrome coronavirus 2 (SARS-CoV-2) belongs to the *Betacoronavirus* genus of the family *Coronaviridae*, which includes enveloped viruses with a single-strand, positive-sense RNA genome (1, 2). Its diameter is approximately 65 to 125 nm. SARS-CoV-2 consists of the following four major structural proteins: spike (S), envelope (E), membrane (M), and nucleocapsid (N). As with other RNA viruses, the genomic RNA replication, mRNA transcription, and protein synthesis of coronavirus occur in the cytoplasm (3). The newly synthesized structural proteins and the RNA genome are assembled into virions, and M drives the virions to bud into the lumen of the endoplasmic reticulum-Golgi intermediary compartment (ERGIC) for further modification and maturation (3, 4). M oligomerization of mouse hepatitis virus (MHV) mediated by its transmembrane domain is hypothesized to enable the formation of a lattice of M (5). S and E were integrated into the lattice by interacting with M (6). Unlike with MHVs, where E and M proteins coexpressed in cells are necessary and sufficient for virus-like particle (VLP) release, M/E/N of SARS-CoV are all required for efficient assembly and release; however, the mechanisms are poorly understood (7–9). Virion release is an essential step in the release of the enveloped virus particles, and the process ultimately triggers the separation of virion and host membranes. VLP systems can be used to determine the individual roles of different viral and cellular proteins in virion release and may be used for vaccine development. Therefore, by using this convenient method to screen and identify the key cellular proteins that function in virion release, we can provide new insights into the details of viral maturation and advance the understanding of the virus-cell interactions.

Ubiquitin modification plays key roles in virus replication. Viruses have evolved to evade immune responses by subverting the host ubiquitin system (10). Ubiquitin is enriched in retrovirus particles, and a variable fraction of the major retroviral structural protein (Gag) is ubiquitinated (11, 12). Additionally, since the ubiquitination of cellular transmembrane proteins can signal the recruitment of class E machinery (vacuolar protein sorting [VPS]), a popular model proposes that the deposition of ubiquitin on viral structural proteins mediates class E machinery recruitment (13). Viral matrix proteins generally contain core consensus amino acid motifs called late (L) domains, which are essential for efficient viral release (14). A study conducted on parainfluenza virus reported that ubiquitination was required for M-mediated VLP production (15, 16), and proteasome inhibitor treatments can also block the viral release of PIV5, Nipah virus (NiV), and Sendai virus (SeV) (17–19). Potential ubiquitination of measles virus (MeV) M has been observed in cells, and endosomal sorting complex required for transport (ESCRT) factors, such as ALG-2 interacting protein X (ALIX), can bind to ubiquitin and enhance viral release (20, 21). Several E3 ligases that regulate viral replication and release have been identified thus far. For example, the HECT-E3 ligase family members interact with viral proteins to regulate the release of mature viral particles: the PPXY motifs in proteins of viruses such as vesicular stomatitis virus (VSV) (22), Ebola virus (23, 24), Rous sarcoma virus (RSV) (25, 26), human T-cell leukemia virus (11, 27–29), and Mason Pfizer monkey virus (30) have been reported bind to various HECT-E3 ubiquitin ligases, including Nedd4, LDI-1, 2, BUL1, WWP1, WWP2, and Itch. The tripartite motif (TRIM) family of E3 ubiquitin ligases also interacts with viral proteins to regulate viral replication: TRIM6 ubiquitinates Ebola virus VP35 to promote replication (31), TRIM69 ubiquitinates dengue virus (DENV) NS3 to inhibit replication (32), and TRIM26 ubiquitinates HCV NS5B to promote replication (33). SARS-CoV-2 uses the host ubiquitin system to polyubiquitinate accessory protein ORF7a at Lys119, and this process enhances ORF7a-mediated inhibition of type I interferon (IFN-I) signaling via STAT2 phosphorylation (34). Thus far, much remains unknown about the utilization of the ubiquitin system for assembly and release by SARS-CoV-2, and the E3 ligase and the deubiquitinating enzyme that play crucial roles have not been determined.

The RING finger protein family (RNF) has been demonstrated to play a role in the regulation of antiviral responses (35–37). RNF5, also known as RMA1, is a RING finger protein and a membrane-anchored (endoplasmic reticulum [ER] and/or mitochondrion) E3 ubiquitin ligase, which is anchored to the ER membrane through a single transmembrane domain spanning the domain located within the C-terminal region. It is implicated in ER-associated protein degradation (ERAD), cell motility, and also negative regulation of autophagy and ER stress (38–41). Several studies have shown the association between RNF5 and the antiviral response: RNF5 negatively regulates virus-triggered signaling by targeting stimulator of interferon genes (STING) and mitochondrial antiviral signaling protein (MAVS) for ubiquitination and degradation in mitochondria (42, 43). Newcastle disease virus V protein degrades MAVS by recruiting RNF5 to polyubiquitinate MAVS (44). Thus far, limited knowledge exists on the role of RNF5 and its potential function as an E3 ligase for viral proteins in the regulation of viral release.

POH1/Rpn11/PSMD14 is a deubiquitinating enzyme within the 19S particle lid that regulates proteasomal activities (45). POH1 functions in various biological processes, including cell differentiation (46), DNA break responses (47), aggresome disassembly (48), and E2F1 stability and tumor formation (49). POH1 was also found to reverse K63- and K11-linked polyubiquitination of E2F1 for tumor formation (49). However, whether POH1 regulates virus production has not been determined.

In this study, we identify RNF5 and POH1 as E3 ligases and deubiquitinating enzymes of SARS-CoV-2 M by using RNA interference (RNAi) screening. A mechanistic study demonstrates that RNF5 regulates virion release by enhancing the interaction of M with E. Furthermore, we show that M targets autophagosomes, and the processes are dependent on RNF5-mediated ubiquitin modification. Overall, our findings reveal the formation and regulation mechanisms of SARS-CoV-2 VLP assembly and release and provide molecular details of the regulation of SARS-CoV-2 virion release. We identify the role played by RNF5 as an E3 ligase for ubiquitination of M, and this will be helpful in the development of novel therapeutic approaches.

## RESULTS

### E and M of SARS-CoV-2 form complexes to mediate VLP assembly and budding.

**FIG 1:**
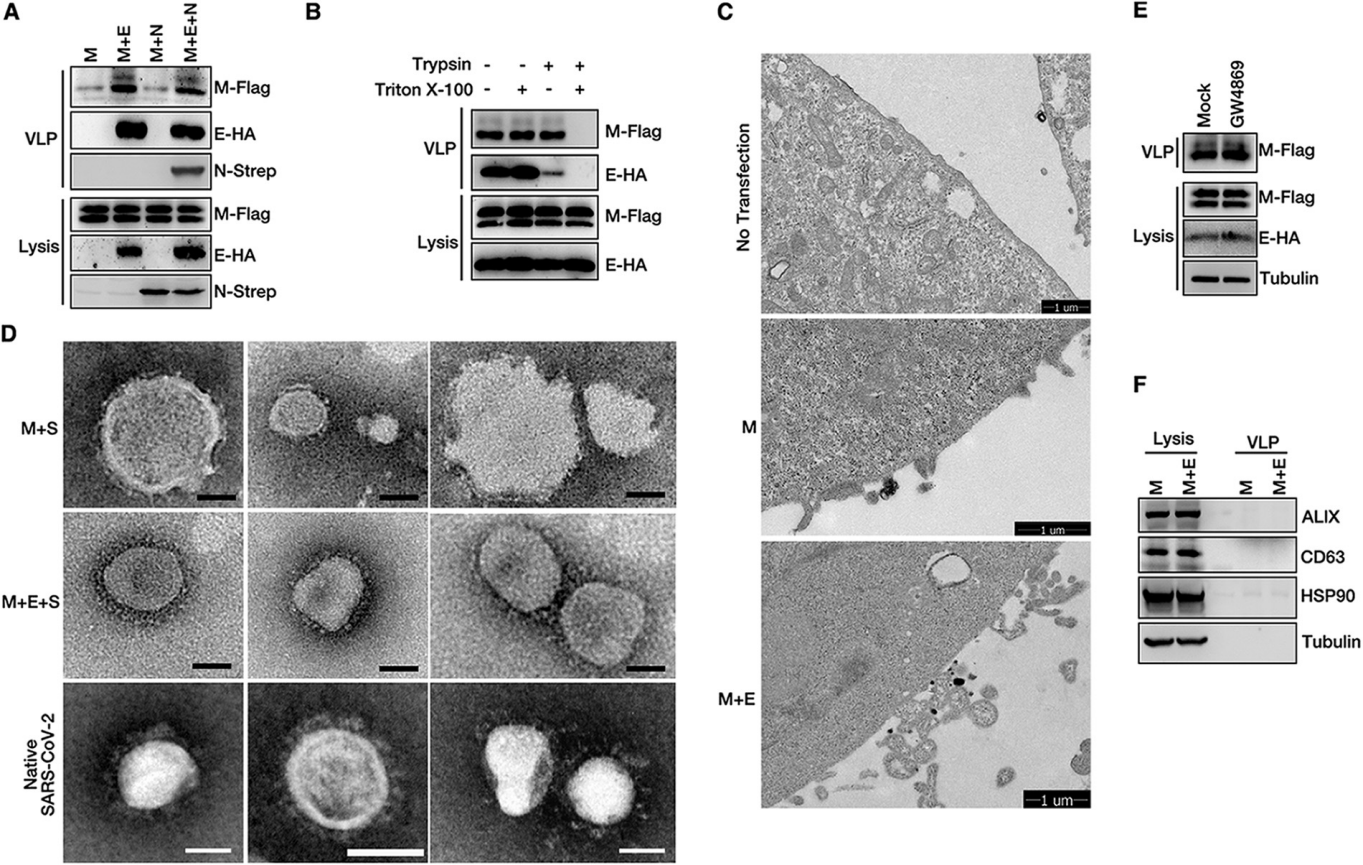
SARS-CoV-2 M and E mediate VLP release. (A) M expression alone and M/E, M/N, or M/E/N coexpression in HEK293T cells. The VLP release assay was performed as described in Materials and Methods and analyzed via Western blotting (WB). (B) HEK293T cells were transfected with SARS-CoV-2 M-Flag and E-HA for 36 h. A protease protection assay of VLPs was performed as described in Materials and Methods, and then the VLPs were analyzed via WB. (C) Representative transmission electron microscopy (TEM) graphs of VLP release in no-transfection cells, cells expressing M alone, or M/E coexpression cells. (D) Representative TEM graphs of SARS-CoV-2 M and S VLPs (top), M, E, and S VLPs (middle), and native SARS-CoV-2 virions (bottom). VLP and virus samples were prepared and then visualized by TEM. Scale bar, 50 nm. (E) HEK293T cells were mock treated or treated with the exosome inhibitor GW4869, and VLP production was analyzed. (F) VLP samples with M expression alone and M/E coexpression were analyzed via WB for exosome-related proteins ALIX, CD63, and HSP90.

The mechanisms of SARS-CoV-2 assembly and release using VLPs were investigated, as VLP systems are reportedly useful tools for studying the viral assembly and release processes of many enveloped viruses. First, transient expression of M alone and of M/E, M/N, and M/E/N of SARS-CoV-2 were achieved, and the culture medium was collected and subjected to ultracentrifugation to pellet VLPs. We found that only M/E or M/E/N coexpression resulted in VLP production; the use of M alone or M/N coexpression did not exhibit such an occurrence, and N expression had no effect on M/E-mediated VLP production (Fig. 1A). To further confirm that the pellet VLPs were indeed the membrane-bound VLPs, VLPs were subjected to treatments with trypsin and/or Triton X-100. No significant digestion of M was observed either in treatments involving the use of trypsin or in those involving Triton X-100; in contrast, under conditions of trypsin plus Triton X-100 treatment, M was completely degraded (Fig. 1B). Furthermore, we used transmission electron microscopy (TEM) to visualize VLP egress directly. The images showed that M/E coexpression, but not M alone, resulted in VLP egress (Fig. 1C). Purified VLPs were negatively stained and analyzed via TEM to determine their morphology and show actual VLPs. The diameter of SARS-CoV-2 virions is approximately 65 to 125 nm. To confirm that what we observed via TEM was VLPs, not exosomes, we coexpressed M with S or with M, E, and S to incorporate the S protein into particles and detected the “crown”-like VLPs. Most particles from M with S were broken and irregular circles and showed inhomogeneous sizes (Fig. 1D, top). As with native SARS-CoV-2 virions, VLPs attributed to M, E, and S coexpression showed a uniform size and displayed a typical coronavirus morphology (Fig. 1D, middle and bottom). Exosome inhibitor GW4869 treatment had no effect on VLP production (Fig. 1E). We also carefully examined exosome-related proteins ALIX, CD63, and HSP90 in the VLP fraction; the result showed that there are very few exosomes in our VLP fraction (Fig. 1F). These data suggest that SARS-CoV-2 E is necessary for M-mediated VLP release. We then used this convenient assay to investigate the mechanisms of SARS-CoV-2 release.

### SARS-CoV-2 M interacts with E, which is required for virion release.

**FIG 2:**
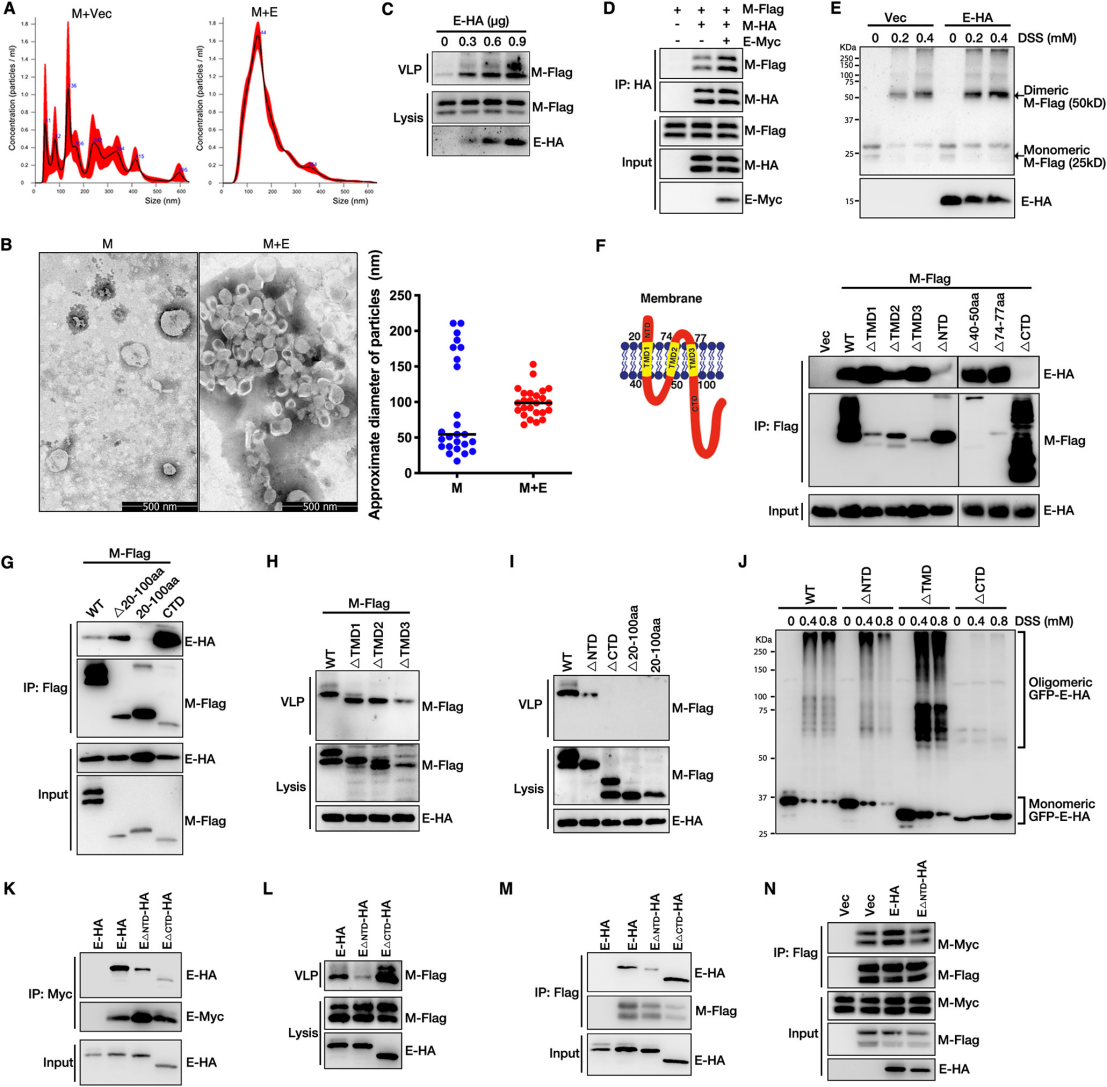
SARS-CoV-2 M interacts with E to mediate viral release. (A) HEK293T cells were transfected with M-Flag with or without E-HA for 36 h, and then the sizes of purified VLPs were analyzed with a NanoSight NS300 (Malvern). Vec, vector. (B) Representative TEM graphs of SARS-CoV-2 VLPs from cells expressing M alone and M/E coexpression cells. The approximate diameters of 25 particles in each case were analyzed. (C) The kinetics of E expression paralleled the increase in M-mediated VLP production. HEK293T cells were transfected as indicated for 36 h, and the VLP release assay was performed and then analyzed via WB. (D) E enhanced M self-interaction. HEK293T cells were transfected with M-HA, M-Flag, and E-Myc for 36 h, subjected to HA IP, and analyzed via WB. (E) E expression enhanced the homo-oligomerization of M. HEK293T cells were transfected with M-Flag and E-HA for 36 h, cells were cross-linked by being treated with disuccinimidyl suberate (DSS) for 30 min, and then lysates were analyzed via WB. (F) Schematic diagrams of wild-type M. HEK293T cells were transfected with E-HA and M-Flag or its mutants for 36 h, subjected to Flag IP, and analyzed via WB to determine the interaction between E and M deletion mutants. (G) HEK293T cells were transfected with E-HA and M-Flag or its mutants for 36 h, subjected to Flag IP, and analyzed via WB. (H) HEK293T cells were transfected with E-HA and M-Flag or M with a deletion of TMD1, TMD2, or TMD3 for 36 h, and the VLP budding assay was performed and analyzed via WB. (I) HEK293T cells were transfected with the indicated plasmids for 36 h. Lysates and the corresponding purified VLPs were analyzed via WB. (J) Homo-oligomerization of wild-type E and its deletion mutants were analyzed. HEK293T cells were transfected with wild-type E or mutant GFP-E-HA for 36 h, and then cells were cross-linked by treatment with DSS for 30 min. Lysates were analyzed via WB. (K) The self-interaction of E was analyzed. HEK293T cells were transfected with E-Myc and GFP-E-HA or its mutants for 36 h, subjected to Myc IP, and analyzed via WB. (L) VLP production was analyzed in wild-type or mutant E expression cells. HEK293T cells were transfected with M-Flag, GFP-E-HA, and mutants for 36 h. Lysates and the corresponding purified VLPs were analyzed via WB. (M) The interaction of M and E was analyzed. HEK293T cells were transfected with M-Flag and GFP-E-HA or its mutants for 36 h, subjected to Flag IP, and analyzed via WB. (N) The self-interaction of M in wild-type or mutant E expression cells was analyzed. HEK293T cells were transfected with M-Flag, M-Myc, GFP-E-HA, and mutant E for 36 h. Lysates were subjected to Flag IP and then analyzed via WB.

We then attempted to decipher the mechanisms by which E promotes M-mediated virion release. We first used NanoSight NS300 (Malvern) to analyze the size distribution of VLPs and found that the size of VLPs attributed to M alone was inhomogeneous, with diameters ranging from 40 nm to 600 nm, and showed several peaks (Fig. 2A), suggesting that M alone failed to efficiently release as complete VLPs. The sizes of VLPs attributed to M-E coexpression were uniform, with an average diameter of 144 nm (Fig. 2A). Purified VLP fractions from M expression alone and M-E coexpression were also analyzed via TEM to determine their morphology; the approximate diameters of 25 particles in each case were analyzed. As shown in Fig. 2B, as with NanoSight NS300 data, the sizes of particles attributed to M-E coexpression were uniform, suggesting that E provided assistance to M to ensure the uniform size of viral particles. The kinetics of E expression paralleled the increase in M-mediated VLP formation (Fig. 2C), suggesting that E enhanced M-mediated VLP release. E enhanced M self-interaction in a coimmunoprecipitation assay (Fig. 2D). In the presence of the cross-linker disuccinimidyl suberate (DSS), we found that M existed in monomeric, dimeric, and oligomeric states and that E expression enhanced the homo-oligomerization of M (Fig. 2E). These results demonstrate a critical role for SARS-CoV2 E in viral M homo-oligomerization. As with the features observed in SARS-CoV, M interacted with E (Fig. 2F). The C-terminal domain (CTD) of M was necessary for the establishment of its interaction with E, as mutant M lacking its CTD (M_ΔCTD_) failed to bind to E (Fig. 2F). M_ΔNTD_ (where NTD is the N-terminal domain) showed a smaller extent of interaction with E (Fig. 2F). The CTD of M alone was sufficient to interact with E (Fig. 2G), suggesting that M interacts with E via its CTD and that the NTD of M is also necessary for the occurrence of the M-E interaction. We then evaluated the ability of VLPs to form these mutants. Surprisingly, deletion of either one of the three transmembrane domains (TMDs) of M had a minor impact on the M-E interaction and VLP release (Fig. 2F and H), as deletion of a single TMD is known to affect the topology of the M protein. M_ΔNTD_ showed weak VLP release (Fig. 2I), which might be due to the establishment of its weak interaction with E (Fig. 2F). Remarkably, the M_ΔCTD_, TMD deletion mutant (deletion of amino acids [aa] 20 to 100), and CTD plus NTD deletion mutant (deletion of aa 1 to 19 and 101 to 222) completely lost their ability to form VLPs (Fig. 2I). These results suggest that M uses its CTD for the M-E interaction, which is critical for viral release.

A previous study had shown that SARS-CoV E forms ion channels on the membrane via homo-oligomerization (50). We then aimed to determine whether SARS-CoV-2 E undergoes homo-oligomerization, which plays an important role in viral release. Several truncations were generated, and the localizations of wild-type (WT) E and deletion mutants were analyzed by confocal imaging; no significant localization change was found between various forms of E (see Fig. S2 in the supplemental material). It was further found that E_ΔCTD_ failed to form homo-oligomerization and showed a smaller extent of self-interaction in a coimmunoprecipitation (co-IP) assay (Fig. 2J and K). Surprisingly, E_ΔCTD_ still promoted M-mediated viral release (Fig. 2L), suggesting that E homo-oligomerization was not required for viral release. Remarkably, the E_ΔNTD_ mutant failed to establish interactions with M (Fig. 2M) and lost its ability to promote VLP release (Fig. 2L), suggesting that E-M interaction, not E homo-oligomerization, was essential for viral release. Furthermore, we found that E_ΔNTD_ failed to enhance the self-interaction of M (Fig. 2N). Taken together, these data suggest that E interacts with M to enhance the self-interaction of M, which ensures the uniform size of viral particles and thus promotes virion release.

10.1128/mBio.03168-21.2 FIG S2 Localization of various forms of E. HeLa cells were transfected with pCDNA4.0-EGFP-E-HA, pCDNA4.0-EGFP-EΔNTD-HA, or pCDNA4.0-EGFP-EΔCTD-HA for 24 h. Cells were analyzed via fluorescence. Scale bar, 10 μm. Download FIG S2, TIF file, 0.6 MB. Copyright © 2022 Yuan et al. 2022 Yuan et al. https://creativecommons.org/licenses/by/4.0/ This content is distributed under the terms of the Creative Commons Attribution 4.0 International license.

### RNF5 promotes virion release though enhancing the interaction of M with E.

**FIG 3:**
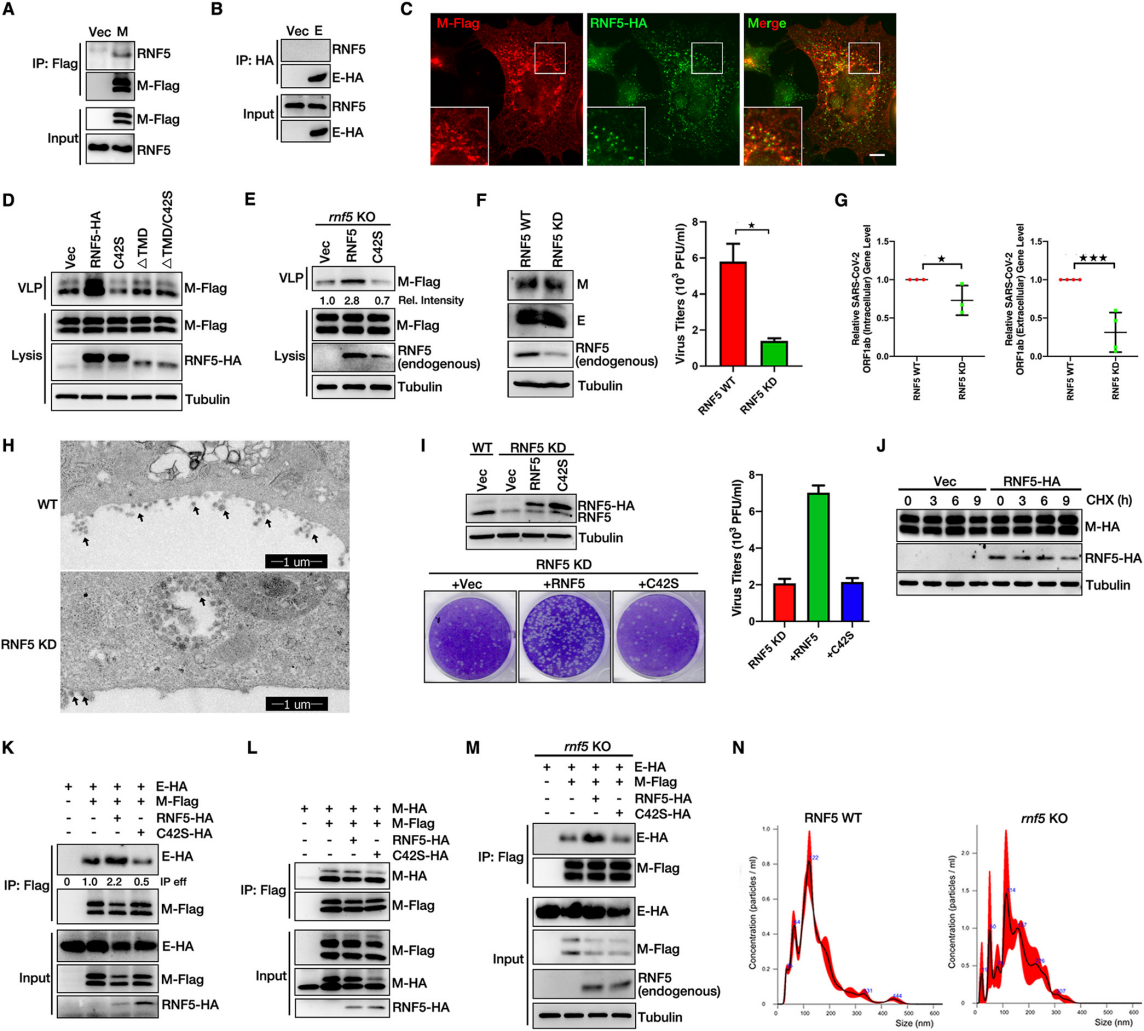
RNF5 promotes the interaction of M with E for viral release. (A and B) RNF5 interacts with M, but not E. HEK293T cells were transfected with M-Flag or E-HA for 36 h. Lysates were subjected to Flag IP or HA IP and analyzed via WB. (C) Colocalization of M and RNF5. AD293 cells were transfected with M-Flag and RNF5-HA for 36 h, and cells were analyzed via immunofluorescence. Flag antibody was used for tracking M, and HA antibody was used for tracking RNF5. Scale bar, 10 μm. (D) Wild-type RNF5, but not the RNF5 C42S, ΔTMD, or ΔTMD/C42S mutant, promotes VLP production. HEK293T cells were transfected with M-Flag, E-HA, and RNF5-HA or with RNF5 C42S-HA, ΔTMD, or ΔTMD/C42S for 36 h. Lysates and the corresponding purified VLPs were analyzed via WB. (E) VLP production was analyzed in rescued *rnf5* KO cells. *rnf5* KO HEK293T cells were transfected with M-Flag, E-HA, and RNF5-HA or RNF5 C42S-HA for 36 h. Lysates and the corresponding purified VLPs were analyzed via WB. (F) WT RNF5 and KD Vero-E6 cells were infected with SARS-CoV-2 (WBP-1) for 24 h, and then media were collected and analyzed via plaque assay as described in Materials and Methods. Cells lysis was analyzed via WB to detect viral M and E protein levels. (G) The levels of intracellular and extracellular SARS-CoV-2 ORF1ab mRNA from the gel in panel F were measured via real-time PCR. Error bars, means ± SD of results from three experiments (*n* = 3). Student's *t* test; *, *P* < 0.05; ***, *P* < 0.001. (H) EM analysis indicates that knockdown of RNF5 decreased the egression of native SARS-CoV-2. Vero cells were infected with SARS-CoV-2 for 36 h and analyzed via TEM. The black arrowheads indicate virion particles. (I) RNF5 KD Vero-E6 cells were transfected with RNAi-resistant WT RNF5 and RNF5 C42S synonymous expression mutants, and then cells were infected with SARS-CoV-2 (WBP-1) for 24 h. Media were collected and analyzed via plaque assay. Cell lysis was analyzed via WB. (J) M-HA-expressing stable HEK293T cells were transfected with or without RNF5-HA and treated with cycloheximide (CHX) for the indicated numbers of hours, and cells lysis was analyzed via WB. (K) RNF5 enhances the interaction of M with E. HEK293T cells were transfected with M-Flag, E-HA, and RNF5-HA or mutant RNF5 C42S-HA for 36 h. Lysates were subjected to Flag IP and analyzed via WB. eff, effect. (L) RNF5 overexpression has no effect on the self-interaction of M. HEK293T cells were transfected with the indicated plasmids for 36 h, subjected to Flag IP, and analyzed via WB. (M) The interaction of M with E was analyzed in rescued *rnf5* KO cells. *rnf5* KO HEK293T cells were transfected with the indicated plasmids for 36 h, subjected to Flag IP, and analyzed via WB. (N) WT and *rnf5* KO HEK293T cells were transfected with M-Flag and E-HA for 36 h, and then the sizes of purified VLPs were analyzed with a NanoSight NS300 (Malvern).

To identify host factors essential for virion release, we performed a small-scale RNAi screening targeting candidates that were on the list from SARS-CoV-2 M immunoprecipitation-mass spectrometry (IP/MS) (51) and found that knockdown (KD) of RNF5 (ring finger protein 5), an ER-localized E3 ligase, significantly reduced virion release (Fig. S1A). We confirmed the interaction of M with RNF5 via co-IP: endogenous RNF5 resulted in coimmunoprecipitation with M, but not with E (Fig. 3A and B). RNF5 colocalized well with M (Fig. 3C). The TMD of RNF5 was critical for the M-RNF5 interaction (Fig. S1B). Overexpression of RNF5 increased VLP release, while its catalytic dead mutant RNF5 C42S reduced VLP release, which might be attributable to the dominant negative effect (Fig. 3D), and RNF5 C42S continued to establish interactions with M (Fig. S1C), suggesting that RNF5 promoted virion release and that this was dependent on E3 ligase activity. To further confirm that the ligase is the only mechanism by which RNF5 promotes VLP assembly, we coexpressed M/E with RNF5 TMD deletion mutants or a C42S mutant lacking the TMD; the results showed that overexpression of RNF5 TMD deletion mutants or the C42S mutant lacking the TMD did not interfere with VLP production (Fig. 3D). Furthermore, to exclude potential off-target effects of small interfering RNA (siRNA), we performed rescue experiments using *rnf5* knockout (KO) cells and found that wild-type RNF5, but not the C42S mutant, rescued the reduction of VLP release (Fig. 3E). Next, we investigated whether RNF5 modulated the maturation and release of native SARS-CoV-2. Wild-type and RNF5 KD Vero cells were infected with SARS-CoV-2 with a multiplicity of infection (MOI) of 0.05, and a plaque assay was subsequently performed. Knockdown of RNF5 had no effect on viral M or E protein intracellular expression (Fig. 3F). Extracellular viral production was lower in RNF5 KD cells than that in wild-type cells (Fig. 3F and Fig. S1D). To exclude the possibility of an infectibility defect in RNF5 KD cells, we evaluated viral gene expression in the extracellular and intracellular regions via real-time PCR and found that intracellular SARS-CoV-2 ORF1ab gene expression was slightly decreased and that extracellular ORF1ab expression was significantly decreased in RNF5 KD cells compared to that observed in wild-type cells (Fig. 3G). We further used TEM to visualize virion egress directly. The images showed that knockdown of RNF5 decreased the egression of SARS-CoV-2 compared to that of wild-type cells (Fig. 3H). Furthermore, we generated RNAi-resistant WT RNF5 and RNF5 C42S synonymous-expression mutants and performed rescue experiments with RNF5 KD Vero cells. Our data showed that only wild-type RNF5, not RNF5 C42S, rescued the reduction of SARS-CoV-2 virus release (Fig. 3I). Taken together, our results suggest that RNF5 facilitates the release of wild-type SARS-CoV-2.

10.1128/mBio.03168-21.1 FIG S1 SARS-CoV-2 M interacts with RNF5 to mediate viral release. (A) HEK293T cells were transfected with the indicated siRNAs and further transfected with E-HA and M-Flag for 36 h, and then cell lysates and the corresponding purified VLPs were analyzed via WB. (B) HEK293T cells were transfected with M-Flag and RNF5-HA or its mutant for 36 h, subjected to Flag IP, and analyzed via WB. (C) HEK293T cells were transfected with the indicated plasmids for 36 h, subjected to Flag IP, and analyzed via WB. (D) WT RNF5 and RNF5 KD Vero-E6 cells were infected with SARS-CoV-2 (WBP-1) for 24 h, and then media were collected and analyzed via plaque assay. (E) M and E can be ubiquitinated. HEK293T cells were transfected with M-Flag or E-Flag for 36 h, subjected to Flag IP, and analyzed via WB. (F) RNF5 overexpression has no effect on the ubiquitin level of E. HEK293T cells were transfected with E-Flag, HA-Ub, and RNF5-HA for 36 h. Lysates were subjected to Flag IP and analyzed via WB. (G) HEK293T cells were transfected with HA-Ub and M-Flag or its mutant for 36 h, subjected to Flag IP, and analyzed via WB. Download FIG S1, TIF file, 1.1 MB. Copyright © 2022 Yuan et al. 2022 Yuan et al. https://creativecommons.org/licenses/by/4.0/ This content is distributed under the terms of the Creative Commons Attribution 4.0 International license.

Next, we aimed to determine whether RNF5 regulated the M-E interaction. Overexpression of RNF5 had a less remarkable impact on the protein levels of M (Fig. 3J). Wild-type RNF5, but not the C42S mutant, increased the M-E interaction (Fig. 3K) but had no effect on M self-interaction (Fig. 3L), suggesting that RNF5 enhanced the interaction of M with E, which was dependent on its E3 ligase activity. Similar results were obtained in rescue experiments with *rnf5* KO cells: wild-type RNF5, but not RNF5 C42S, rescued the M-E interaction (Fig. 3M). We further used NanoSight NS300 (Malvern) to analyze the size distribution of VLPs from *rnf5* KO cells and found that knockout of *rnf5* led to the generation of inhomogeneous VLPs, suggesting that RNF5 ensured the uniform size of viral particles (Fig. 3N). Taken together, these results suggest that RNF5 enhances the interaction of M with E to ensure the uniform size of viral particles.

### Ubiquitination of SARS-CoV-2 M mediated by RNF5 is critical for efficient virion release.

**FIG 4:**
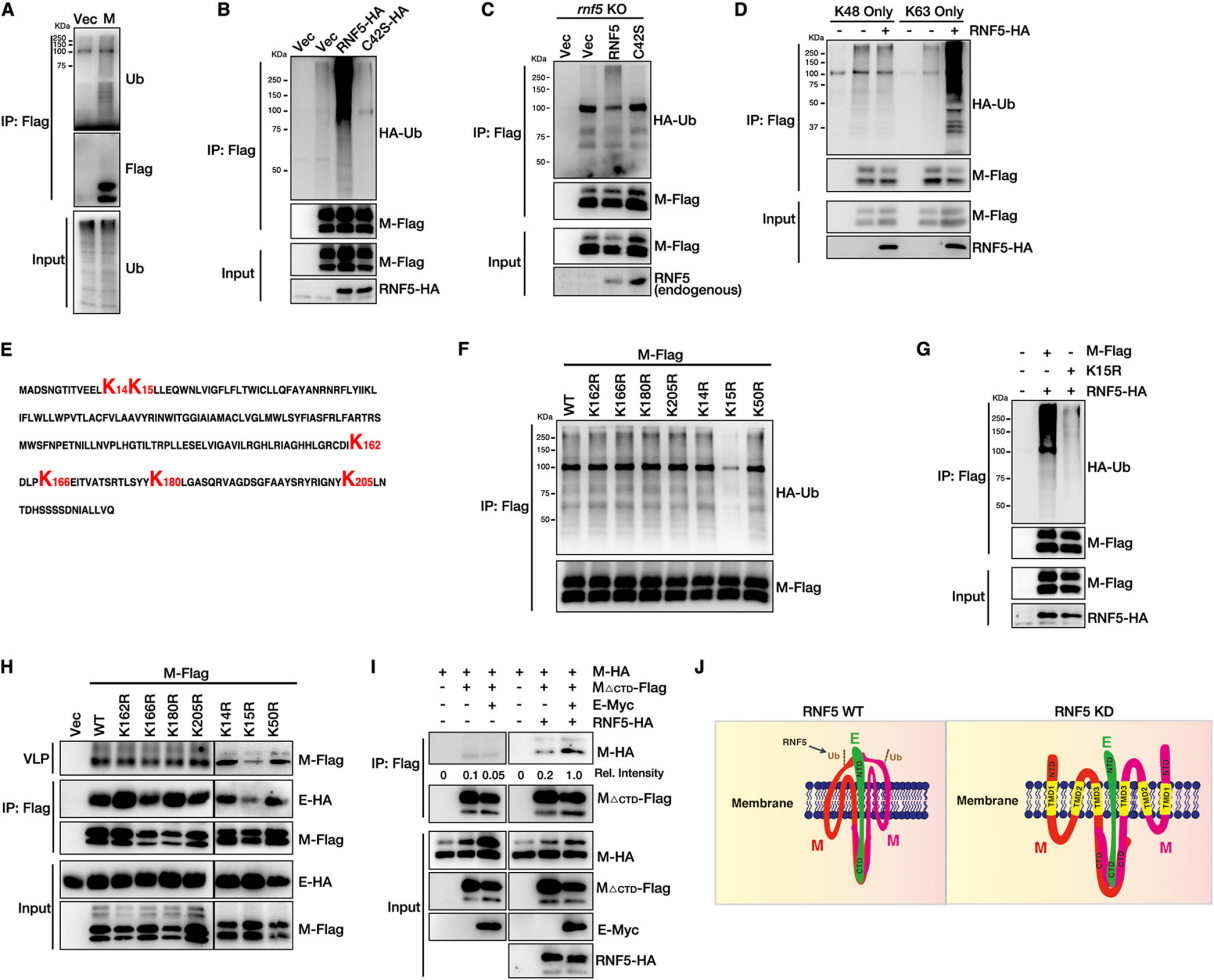
The ubiquitination of SARS-CoV-2 M mediated by RNF5 is critical for viral release. (A) M can be ubiquitinated (Ub). HEK293T cells were transfected with M-Flag for 36 h, subjected to Flag IP, and analyzed via WB. (B) RNF5 overexpression enhances the ubiquitin level of M. HEK293T cells were transfected with M-Flag, HA-Ub, and RNF5-HA or mutant RNF5 C42S-HA for 36 h. Lysates were subjected to Flag IP and analyzed via WB. (C) The ubiquitin level of M was analyzed in rescued *rnf5* KO cells. *rnf5* KO HEK293T cells were transfected with M-Flag, HA-Ub, and RNF5-HA or RNF5 C42S-HA for 36 h. Lysates were subjected to Flag IP and analyzed via WB. (D) HEK293T cells were transfected with M-Flag with HA-UbK48 only or HA-UbK63 only with or without RNF5-HA for 36 h. Lysates were subjected to Flag IP and analyzed via WB. (E) Amino acid sequence of SARS-CoV-2 M. (F) K15 is the main ubiquitin site of M. HEK293T cells were transfected with M-Flag or mutant M proteins with HA-Ub. Lysates were subjected to Flag IP and analyzed via WB. (G) RNF5 overexpression failed to enhance the ubiquitin level of mutant M K15R. HEK293T cells were transfected with M-Flag or M K15R-Flag, HA-Ub, and RNF5-HA for 36 h. Lysates were subjected to Flag IP and analyzed via WB. (H) HEK293T cells were transfected with the indicated M mutants and E-HA, lysates were subjected to Flag IP to detect the interaction of M with E, and the corresponding purified VLPs were analyzed via WB. (I) RNF5 promotes the interaction of M’s NTD with E. HEK293T cells were transfected with the indicated plasmids for 36 h, subjected to Flag IP, and analyzed via WB. (J) Models of RNF5 regulating the M-E interaction.

RNF5 is an E3 ligase that enhances the interaction between M and E, which is dependent on its E3 ligase activity. Thus, we speculated that RNF5 ubiquitinated M to regulate M-E interaction and virion release. We first confirmed that both M and E could be ubiquitinated (Fig. 4A and Fig. S1E). Overexpression of wild-type RNF5, but not RNF5 C42S, enhanced the ubiquitination level of M (Fig. 4B). Overexpression of RNF5 has no effect on the ubiquitination level of E (Fig. S1F). We also performed rescue experiments in *rnf5* KO cells and found that knockout of *rnf5* completely abolished the ubiquitination of M and that wild-type RNF5, but not RNF5 C42S, rescued the ubiquitination of M (Fig. 4C). Overexpression of RNF5 only increased K63-linked polyubiquitination and had a minor effect on the K48-linked polyubiquitination of M (Fig. 4D). These results suggest that RNF5 acts as an E3 ligase for K63-linked ubiquitination of M. We next sought to define the critical lysine residues of ubiquitin modification in M, which might be responsible for the release of VLPs. M_ΔNTD_ completely lost its ubiquitination ability (Fig. S1G). Two lysine repeats, K14/K15, are localized to the N terminus, and four lysine repeats, K162/K166/K180/K205, are localized to the C terminus of M (Fig. 4E). We found that only residue K15 was mainly responsible for M ubiquitination, as the K15R mutant completely abolished the ubiquitin modification (Fig. 4F). Furthermore, RNF5 failed to enhance the ubiquitination level of the K15R mutant (Fig. 4G). These results indicate that residue K15 is the main ubiquitin modification site in M. Ubiquitination modification can alter its localization or binding partners. Next, we aimed to determine whether ubiquitination in M played a potential role in the M-E interaction and SARS-CoV-2 virion release. To verify this assumption, we performed co-IP and VLP assays. Notably, only the K15R mutant showed less M-E interaction and a remarkable reduction in VLP release (Fig. 4H). These data suggest that RNF5 ubiquitinates M at the K15 residue to enhance the interaction between M and E.

We showed that (i) M used its CTD for the M-E interaction, whereas deletion of the NTD from M decreased the M-E interaction and viral release (Fig. 2F and H), and (ii) the ubiquitin modification site was located in the NTD of M (Fig. 4F), which was critical for both the M-E interaction and viral release. Based on these results, we speculated that RNF5 ubiquitinated M at the K15 residue to enhance the interaction of M’s NTD with E to increase the stability of the M-E complex on the membrane and ensure the uniform size of VLPs for viral maturation (Fig. 4J). To verify this hypothesis, we examined the self-interaction of M_ΔCTD_ with or without E and RNF5 via co-IP. The result indicates that M_ΔCTD_ alone showed less self-interaction and coexpression of E and that RNF5 significantly increased the self-interaction of M_ΔCTD_ (Fig. 4I). Taken together, these results indicate that E binds to the CTD of M and promotes the homo-oligomerization of M; RNF5 ubiquitinates M at the K15 residue to enhance the interaction of M’s NTD with E, thus enhancing the stability of the M-E complex to promote virion assembly and release (Fig. 4J).

### POH1 deubiquitylates SARS-CoV-2 M to inhibit virion release.

**FIG 5:**
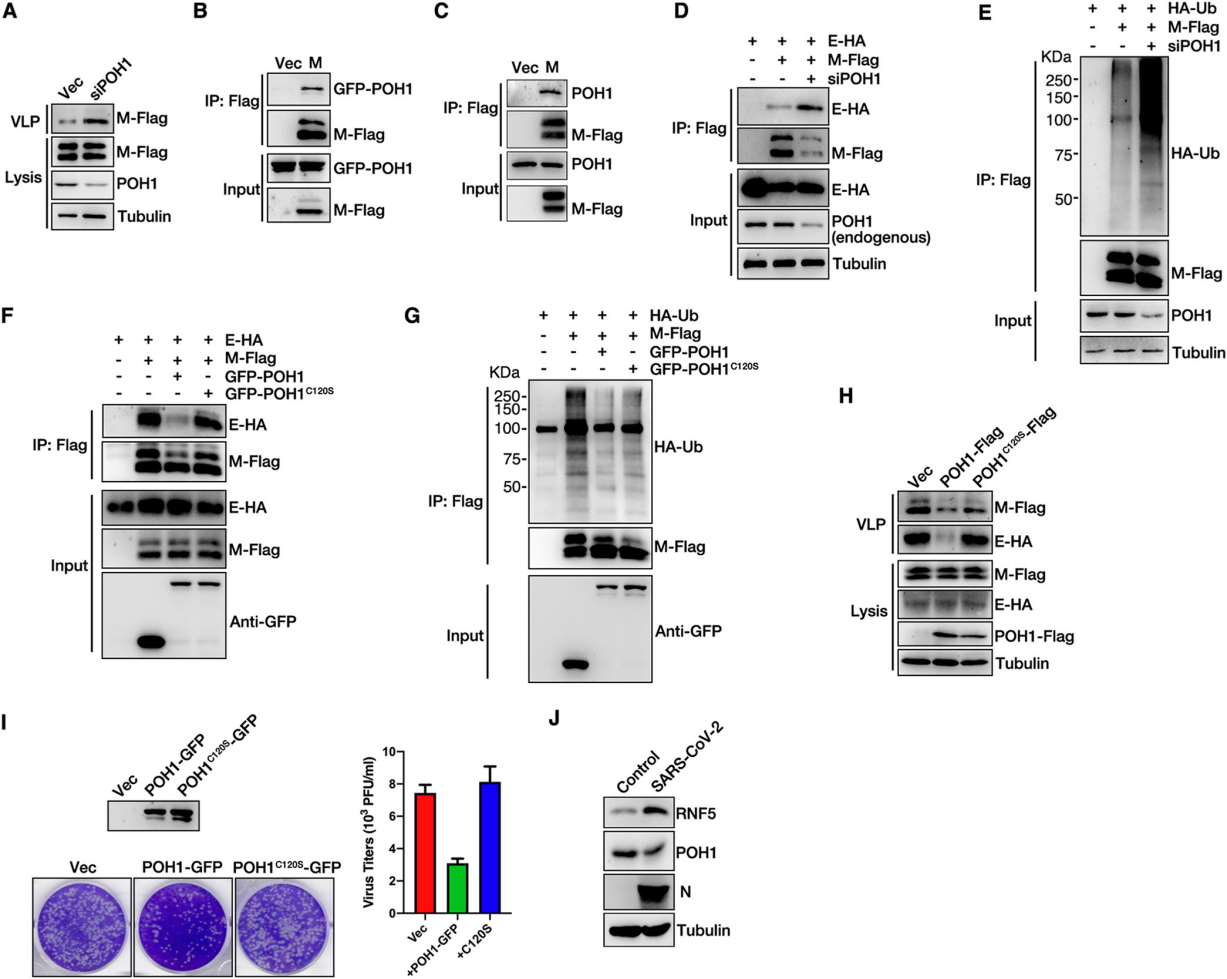
POH1 deubiquitinates M and inhibits viral release. (A) The knockdown of POH1 promotes VLP production. HEK293T cells were transfected with E-HA and M-Flag, with or without POH1 siRNA, for 36 h. Lysates and the corresponding purified VLPs were analyzed via WB. (B) M interacts with GFP-POH1. HEK293T cells were transfected with GFP-POH1 and M-Flag for 36 h, subjected to Flag IP, and analyzed via WB. (C) M interacts with endogenous POH1. HEK293T cells were transfected with M-Flag for 36 h, subjected to Flag IP, and analyzed via WB. (D) The knockdown of POH1 enhances the interaction of M with E. HEK293T cells were transfected with POH1 siRNA, further transfected with E-HA and M-Flag for 36 h, subjected to Flag IP, and analyzed via WB. (E) The knockdown of POH1 enhances the ubiquitin level of M. HEK293T cells were transfected with POH1 siRNA, further transfected with HA-Ub and M-Flag for 36 h, subjected to Flag IP, and analyzed via WB. (F) HEK293T cells were transfected with the indicated plasmids, and the interaction of M and E was analyzed in wild-type or C120S mutant POH1 overexpression cells via Flag IP. (G) HEK293T cells were transfected with the indicated plasmids, and the ubiquitin level of M was analyzed in wild-type or C120S mutant POH1 overexpression cells via Flag IP. (H) HEK293T cells were transfected with the indicated plasmids, and VLP production was analyzed in wild-type or C120S mutant POH1 overexpression cells. (I) Vero-E6 cells were transfected with wild-type or C120S mutant POH1 expression mutants, cells were infected with SARS-CoV-2 (WBP-1) for 24 h, and media were collected and analyzed via plaque assay. Cell lysis was analyzed via WB. (J) Vero-E6 cells were infected with or without SARS-CoV-2 (WBP-1) for 24 h. Cells lysis was analyzed via WB to detect SARS-CoV-2 N, RNF5, and POH1 protein levels.

Based on the results obtained from RNAi screening targeting candidates that are present on the list derived from SARS-CoV-2 M IP/MS, we also attempted to identify the proteins that negatively regulate VLP release, especially the deubiquitinating enzyme POH1; knockdown of POH1 increased VLP release (Fig. S1A and Fig. 5A). We speculated that POH1 might act as a deubiquitinating enzyme for M to negatively regulate virion release. To verify this hypothesis, we first confirmed that M interacted with green fluorescent protein (GFP)-POH1 and endogenous POH1 via co-IP (Fig. 5B and C). Next, we explored whether POH1 regulated the ubiquitination of M and M-E interactions. Knockdown of POH1 enhanced the interaction between M and E (Fig. 5D) and the ubiquitination level of M (Fig. 5E). To further confirm that POH1 acts as a deubiquitinating enzyme for M, we analyzed the M-E interaction, ubiquitin level of M, and VLP release in WT POH1 or the overexpression of catalytic dead C120S mutant cells. Our data showed that overexpression of wild-type POH1, but not of the catalytic dead C120S mutant, decreases the M-E interaction (Fig. 5F), the ubiquitin level of M (Fig. 5G), and VLP release (Fig. 5H). Next, we investigated whether POH1 modulated the maturation and release of native SARS-CoV-2. WT POH1- or catalytic dead C120S mutant-overexpressing Vero cells were infected with SARS-CoV-2 at an MOI of 0.05, and a plaque assay was subsequently performed. The data showed that overexpression of wild-type POH1, but not of the C120S mutant, caused the reduction of SARS-CoV-2 virus release (Fig. 5I). Taken together, these results indicate that POH1 may act as a deubiquitinating enzyme of M and may negatively regulate virion release by inhibiting the interaction between M and E. We further compared the protein levels of RNF5 and POH1 in control and SARS-CoV-2-infected cells, respectively. Our result showed that the expression of POH1 was decreased but that that of RNF5 was significantly increased in SARS-CoV-2-infected cells compared to levels in control cells (Fig. 5J), suggesting that SARS-CoV-2 decreased the expression of POH1 and increased the expression of RNF5 to ensure the ubiquitin modification of M and viral release.

### SARS-CoV-2 M uses autophagosomes for virion release.

**FIG 6:**
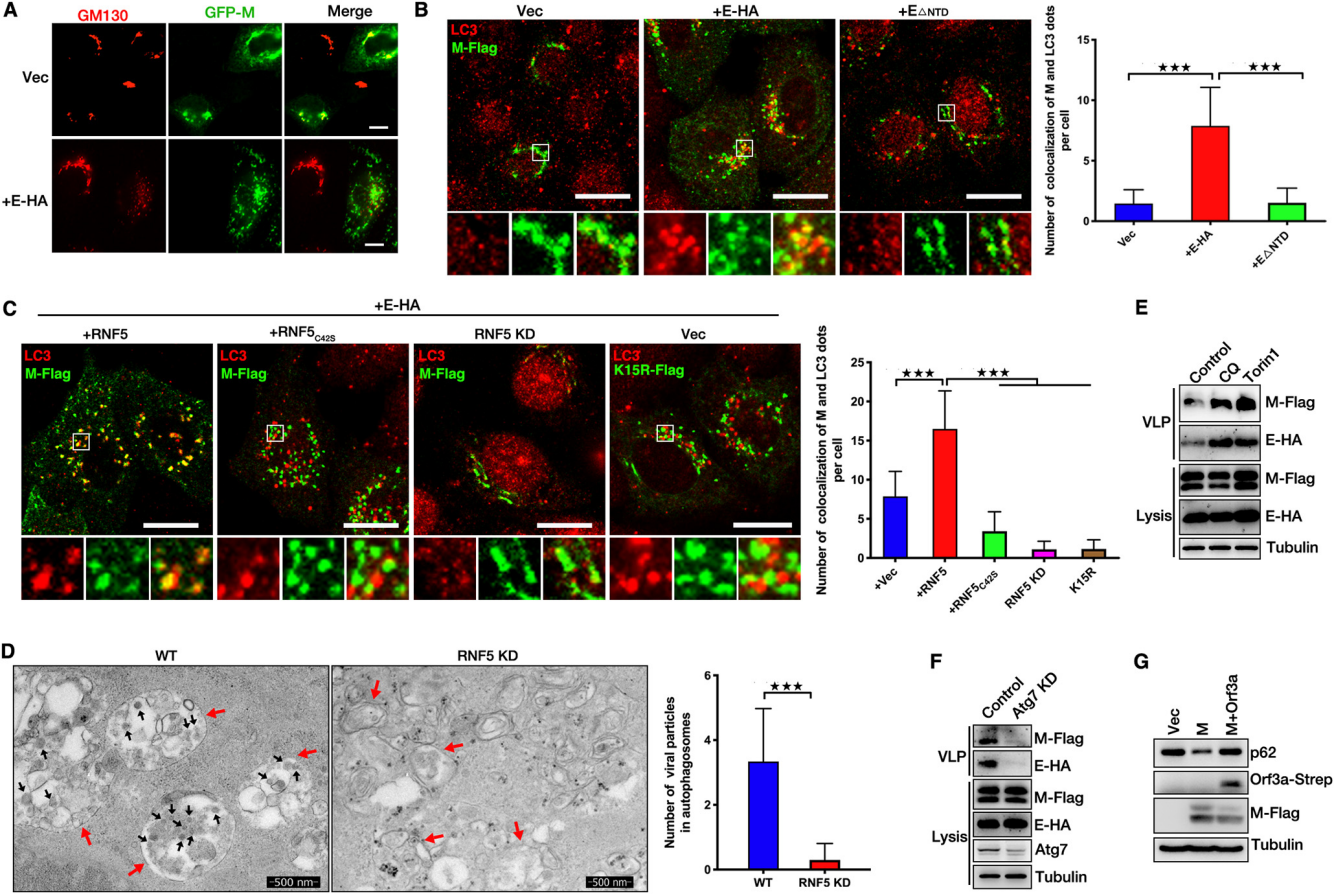
RNF5 promotes the trafficking of SARS-CoV-2 M from the Golgi apparatus to autophagosomes for virions release. (A) HeLa cells were transfected with GFP-M with or without E-HA for 24 h. Cells were analyzed via immunofluorescence. GM130 antibody was used for tracking the Golgi apparatus. Scale bar, 10 μm. (B) HeLa cells were transfected with M-Flag and E-HA or E_ΔNTD_ for 24 h, and cells were analyzed via immunofluorescence. Flag antibody was used for tracking M, and LC3 antibody was used for tracking autophagosome. Scale bar, 10 μm. The graphs show the quantification of numbers of cells colocalizing with M and LC3; the numbers of dots per cell were determined by taking the average number of dots in 50 cells. Error bars, means ± SD from three experiments. Student's *t* test; ***, *P* < 0.001. (C) HeLa cells were transfected with M-Flag and E-HA with or without wild-type or C42S mutant RNF5 for 24 h or siRNF5 for 48 h, and cells were analyzed via immunofluorescence. Flag antibody was used for tracking M, and LC3 antibody was used for tracking autophagosome. Scale bar, 10 μm. The graphs show the quantification of numbers of cells colocalizing with M and LC3; the numbers of the indicated dots per cell were determined by taking the average number of dots in 50 cells. Error bars, means ± SD from three experiments. Student's *t* test; ***, *P* < 0.001. (D) Electron micrograph analysis indicates that knockdown of RNF5 abolished the accumulation of virion particles in autophagosomes. Vero cells were infected with SARS-CoV-2 for 36 h and analyzed via TEM. The black arrows indicate virion particles, and red arrows indicate autophagosomes. The graphs show the quantification of numbers of virion particles and autophagosomes that colocalized; numbers were determined by counting the average number of virion particles in 50 autophagosomes. Error bars, means ± SD from three experiments. Student's *t* test; ***, *P* < 0.001. (E) HEK293T cells were transfected with E-HA and M-Flag for 30 h. Cells were further treated with CQ or Torin1 for another 6 h. Lysates and the corresponding purified VLPs were analyzed via WB. (F) HEK293T cells were transfected with E-HA and M-Flag with or without Atg7 siRNA for 36 h. Lysates and the corresponding purified VLPs were analyzed via WB. (G) HEK293T cells were transfected with M-Flag with or without Orf3a-Strep for 36 h, and cell lysis was analyzed via WB.

We have discussed the details of SARS-CoV-2 viral assembly and release. Subsequently, we aimed to determine the mechanisms of virion trafficking to the cell membrane. Our previous study indicated that M of HPIV3 colocalized with LC3 and that autophagosomes enhanced the ability of virions to bind to the membrane and release (52). We then explored whether SARS-CoV-2 hijacked autophagosomes for release. We first determined the location of M. M expression alone localized to the Golgi apparatus (GM130) (Fig. 6A). When coexpressed with E, M failed to target the Golgi apparatus (Fig. 6A). M showed a colocalization with autophagosomes when coexpressed with wild-type E, but not E_ΔNTD_ (this mutant fails to interact with M) (Fig. 6B), suggesting that E interacts with M to promote the trafficking of M from the Golgi apparatus to autophagosomes. We further found that (i) overexpression of wild-type RNF5, but not of the C42S mutant, promoted the localization of M to autophagosomes, (ii) M failed to target autophagosomes in RNF5 KD cells, and (iii) the ubiquitin modification defect K15R mutant failed to target autophagosomes (Fig. 6C), suggesting that RNF5 promotes the trafficking of M from the Golgi apparatus to autophagosomes, a process which was dependent on its E3 ligase activity. We further determined the colocalization of virions and autophagosomes via TEM. As for a previous report (53), we found that SARS-CoV-2 virions accumulated in autophagosomes and that knockdown of RNF5 abolished this colocalization (Fig. 6D). Chloroquine (CQ) treatment blocks autophagic degradation and accumulates autophagosomes in cells, and Torin1 treatment inhibits mTORC1 and induces autophagic flux. Both CQ and Torin1 treatment leads to an increased number of autophagosomes in cells. We then treated cells with CQ or Torin1 and subjected them to the VLP assay. Remarkably, CQ or Torin1 treatment enhanced VLP release (Fig. 6E), and knockdown of *Atg7*, the key gene for autophagy induction, reduced the release of VLPs (Fig. 6F), suggesting that autophagosomes promoted SARS-CoV-2 virion release. Previous studies showed that M expression induces autophagy (54), and ORF3a of SARS-CoV-2 blocks the fusion between autophagosomes and lysosomes (53). We also found that ORF3a inhibits the degradation of p62 induced by M expression (Fig. 6G), suggesting that in SARS-CoV-2-infected cells, M uses autophagosomes, not autolysosomes, for release. These results showed that M uses autophagosomes for virion release and that RNF5-mediated ubiquitin modification in both M and E-M is required for the trafficking of M from the Golgi apparatus to autophagosomes. Taken together, our data showed that RNF5-mediated ubiquitin modification in M was critical for SARS-CoV-2 virion release via enhancement of the interaction of M and E and the induction of autophagy.

## DISCUSSION

In this study, we used the VLP system to comprehensively investigate the molecular mechanisms of SARS-CoV-2 release in detail. We showed that SARS-CoV-2 E could bind to the CTD and NTD of M to ensure the uniform size of SARS-CoV-2 viral particles. We further identified RNF5 and POH1 as E3 ligases and deubiquitinating enzymes, respectively, that played crucial roles in the regulation of the interaction of M with E via ubiquitin modification of M. RNF5 ubiquitinates M at the residue K15 to enhance the interaction of its NTD with E, thus enhancing the stability of the M-E complex on the membrane and ensuring the uniform size of VLPs to promote viral release. Knockdown of RNF5 decreases, while overexpression of RNF5 increases, SARS-CoV-2 VLP release. SARS-CoV-2 infection experiments also showed that extracellular viral production (virions released into supernatants) was lower in RNF5 KD cells or POH1 overexpression cells than that in wild-type cells. We also performed rescued experiments in RNF5 KD cells to further confirm that RNF5 facilitates SARS-CoV-2 egress via its E3 ligase activity. We further determined the mechanism by which M mediated virion trafficking to the cell membrane for release. We found that CQ or Torin1 treatment significantly enhanced VLP release and that knockdown of Atg7 significantly decreased VLP release, suggesting a critical role for autophagy in virion release. M traffics from the Golgi apparatus to autophagosomes with coexpression of E, which is dependent on RNF5-mediated ubiquitin modification. All together, these data revealed a previously undescribed mechanism by which RNF5 and POH1 mediate the ubiquitin modification of SARS-CoV-2 M for virion trafficking and release.

As noted by previous studies, unlike with most enveloped viruses, in which M is necessary and sufficient to mediate VLP release, the E/M proteins of murine hepatitis virus (MHV) or the M/E/N proteins of SARS-CoV are necessary for efficient assembly, trafficking, and VLP release (7–9). A previous study has shown that expression of M and E is essential for the efficient formation and release of SARS-CoV-2 VLPs (55). Another study showed that coexpression of M/N/E/S is the most efficient SARS-CoV-2 system for VLP production (56). A recent study suggested that VLP production and secretion were highly dependent on N proteins (57), but the mechanism(s) by which SARS-CoV-2 VLPs are released remains unknown. In the present study, we found that M and E of SARS-CoV-2 were necessary and sufficient to mediate VLP release, while N was not critical for this process. E interacts with M to promote viral release, and E homo-oligomerization is not required for the release of VLPs. The diameter of SARS-CoV-2 is approximately 65 to 125 nm. Viruses use strategies to avoid the production of defective virions, but the mechanism(s) by which viruses can ensure the uniform size distribution of viral particles is poorly understood. Our data suggest that M forms a complex with E on the VLP membrane and that RNF5 enhances the interaction of M and E by ubiquitinating M at K15 to enhance the binding ability of M’s NTD with E. This mechanism may ensure the stability of the M-E complex on a membrane with high surface tension and may help maintain the uniform size of VLPs and avoid the production of defective virions. Further *in vitro* biochemistry experiments and structural analysis of the M-E complex should be performed to elucidate the details of the mechanisms by which the virus can ensure the uniform size distribution of viral particles.

Ubiquitination and the ubiquitin-proteasome system (UPS) are involved in the replication of coronaviruses. The role of cellular ubiquitination, particularly for the E3 ubiquitin ligase Nedd4 and Itch, in promoting the efficient budding of many enveloped RNA viruses, including Ebola virus (EBOV), has been well documented (23, 58). Proteasomal inhibition can also regulate the replication of the MHV, and in the presence of the proteasome inhibitor MG132, the entering viruses accumulate in both the endosome and the denser lysosome, indicating that the ubiquitin-proteasome system is involved in the release of virus from the endosome to the cytosol during the virus entry step (59). However, MHV assembly and release are not appreciably affected by proteasomal inhibition compounds (60). We have shown that the ubiquitin modification of viral protein M is critical for SARS-CoV-2 assembly and release. Furthermore, we noted that certain proteasomal inhibitors, such as MG132, may also inhibit the lysosomal proteins cathepsin B and A (61), and a recent study has reported that SARS-CoV-2 uses lysosomes for egress (53). These studies highlight the critical roles of ubiquitin in the virus life cycle, and it will be of interest to determine whether proteasomal inhibitors, such as MG132, can be used as effective new therapeutic agents for SARS-CoV-2 infection.

RNF5-mediated ubiquitination of M, instead of regulating the stability of M, enhances the interaction of M with E to promote the steady state of the M-E complex. Notably, a mutant, M_K15R_, that is deficient in ubiquitin modification lost its ability to release VLPs, suggesting that RNF5 played a critical role in controlling the release and maturation of SARS-CoV-2. As with our finding that RNF5 regulates protein interaction via ubiquitin modification, RNF5 also interacts with JAMP (JNK1/MAPK8 associated membrane protein), resulting in Lys-63 chain ubiquitination, which did not alter JAMP stability but rather decreased its association with proteasome subunits and p97, a key component of the ERAD response (40). RNF5 has been more widely studied for its role in immune regulation, such as its ability to negatively regulate virus-triggered signaling by targeting STING and MAVS for ubiquitination and degradation in the mitochondria (42, 43). Whether there is any association between the immune-regulatory activities of RNF5 and its role in promoting viral assembly and release remains to be determined, as does the role of RNF5 in facilitating viral release and in modulating the pathogenesis of infectious SARS-CoV-2. Furthermore, our data showed that the expression of POH1 was decreased but that the expression of RNF5 was increased in SARS-CoV-2-infected cells compared to that in control cells (Fig. 5J), suggesting that SARS-CoV-2 decreased the expression of POH1 and increased the expression of RNF5 to ensure the ubiquitin modification of M and viral release. It will be of interest to determine whether SARS-CoV-2 regulates the mRNA or protein levels of RNF5 or POH1 in SARS-CoV-2-infected human tissues.

Autophagy is a multistep process by which cytoplasmic components are engulfed by autophagosomes and shuttled to lysosomes for degradation (62). Viruses have developed strategies to subvert or to directly utilize autophagy for their own replication and survival. For example, our previous study showed that a phosphoprotein of HPIV3 could block autophagosome-lysosome fusion to increase virus production (52). ORF3a of SARS-CoV-2 blocks the homotypic fusion and vacuole protein sorting (HOPS) complex-mediated assembly of the SNARE (soluble *N*-ethylmaleimide-sensitive factor attachment protein receptor) complex required for autolysosome formation (53). A recent study has reported that SARS-CoV-2 uses lysosomes for egress instead of the biosynthetic secretory pathway and has highlighted the critical role of lysosomal exocytosis in virus egress (63). However, the role of autophagosomes as cargo for SARS-CoV-2 release has not been determined. In the present study, we found that M targeted autophagosomes with coexpression of E. RNF5-mediated ubiquitin modification is critical for M-induced autophagy. Further experiments are warranted to elucidate the mechanism by which M induces autophagy and the manner in which autophagosomes promote virion release. Our unpublished data suggest that M does not establish interactions with autophagic proteins, suggesting that M induces autophagy via a new autophagic regulatory factor. Using siRNA screening, we identified a novel candidate that regulates autophagy and establishes interactions with M, which might serve as a key adaptor between autophagy and M.

This study has several limitations. Although wild-type and RNF5 KD Vero cells were infected with SARS-CoV-2 and analysis was performed by conducting plaque assays, for obtaining most conclusions that M-E coexpression mediates virion release in this study, we relied on VLP systems rather than wild-type viruses. As such, further experiments, such as those involving the use of the real recombinant virus to investigate SARS-CoV-2 assembly and release, should be performed to complement the VLP system.

In summary, our results provide new insights into the E3 ligase RNF5, which ubiquitinates and regulates SARS-CoV-2 M-mediated release. Combined with researchers from previous studies, researchers of our group and other groups have determined the following critical roles of RNF5 in virus infection: RNF5 ubiquitinates STING and MAVS for degradation to decrease the IFN-β response, and RNF5 ubiquitinates SARS-CoV-2 M to facilitate virion release. Overall, our findings indicate that RNF5 is a potential target for antiviral drug development.

## MATERIALS AND METHODS

### Cell cultures.

HEK293T, *rnf5* KO HEK293T, AD293, HeLa, and Vero cells were cultured in Dulbecco’s modified Eagle’s medium (DMEM; GIBCO) supplemented with 10% fetal bovine serum (FBS, Sigma-Aldrich) at 37°C with 5% CO_2_.

### Plasmid construction.

pCDNA4.0-M-Flag, pCDNA4.0-MΔTMD1-Flag, pCDNA4.0-MΔTMD2-Flag, pCDNA4.0-MΔTMD3-Flag, pCDNA4.0-MΔNTD-Flag, pCDNA4.0-MΔ40-50aa-Flag, pCDNA4.0-MΔ74-77aa-Flag, pCDNA4.0-MΔCTD-Flag, pCDNA4.0-MΔ20-100aa-Flag, pCDNA4.0-M-CTD-Flag, pCDNA4.0-M-20-100aa-Flag, pCDNA4.0-M-K14R-Flag, pCDNA4.0-M-K15R-Flag, pCDNA4.0-M-K50R-Flag, pCDNA4.0-M-K162R-Flag, pCDNA4.0-M-K166R-Flag, pCDNA4.0-M-K180R-Flag, and pCDNA4.0-M-K205R-Flag were cloned into the mammalian expression vector pCDNA4.0-Flag. pCDNA4.0-M-HA, pCDNA4.0-E-HA, pCDNA4.0-RNF5-HA, pCDNA4.0-RNF5ΔTMD-HA, pCDNA4.0-RNF5-C42S-HA, pCDNA4.0-RNF5ΔTMD-C42S-HA, pCDNA4.0-RNF5 resistant-HA, and pCDNA4.0-RNF5-C42S resistant-HA were cloned into the mammalian expression vector pCDNA4.0-HA. pCDNA3.0-HA-UB, pCDNA3.0-HA-UB K48, and pCDNA3.0-HA-UB K63 were cloned into the mammalian expression vector pCDNA3.0-HA. pCDNA4.0-E-Myc and pCDNA4.0-M-Myc were cloned into the mammalian expression vector pCDNA4.0-Myc. pTY-M-HA was cloned into the mammalian expression vector pTY-HA. pCDNA4.0-EGFP-E-HA, pCDNA4.0-EGFP-EΔNTD-HA, pCDNA4.0-EGFP-EΔTMD-HA, and pCDNA4.0-EGFP-EΔCTD-HA were cloned into the mammalian expression vector pCDNA4.0-EGFP-HA.

### Oligonucleotides.

RNF5, GPAT4, ACSL3, PGAM5, AGPAT4, AUP1, DERL1, AGPAT5, SEL1L, OS9, FAM8A1, DERL2, UBE2J1, UBE2G2, RIINT1, RNF126, RNF138, RNF170, POH1, ZW10, Atg7, and EDEM3 siRNAs were purchased from Guangzhou RiboBio. The sequence of RNF5 single guide RNA (sgRNA) is GAAGGGCCAAATCGCGAGCG.

### Antibodies and reagents.

Mouse monoclonal anti-Flag (F1804) and anti-hemagglutinin (HA) (H3663) were obtained from Sigma-Aldrich. Anti-Myc (2278) and Atg7 (8558S) were obtained from Cell Signaling Technology. Anti-RNF5 (sc-81716), ALIX (sc-53540), and ubiquitin (sc-8017) were obtained from Santa Cruz Biotechnology. Anti-HSP90 (60318-1-Ig) was obtained from Proteintech. Rabbit anti-LC3 (PM036) was obtained from MBL. Anti-tubulin (E7-S) was obtained from the Developmental Studies Hybridoma Bank. POH1 (A9608), CD63 (A19023), SARS-CoV-2 M (A20232), and SARS-CoV-2 E (A20199) were obtained from ABclonal. SARS-CoV-2 N (40143-MM05) was obtained from Sino Biological Inc. Mouse anti-p62 (H00008878-M01) was obtained from Abnova. Goat anti-mouse IgG (H+L) secondary antibody, Alexa Fluor 568 conjugate (A11031), goat anti-rabbit IgG (H+L) secondary antibody, Alexa Fluor 568 conjugate (A11036), goat anti-mouse IgG (H+L) secondary antibody, Alexa Fluor 488 conjugate (A32723), goat anti-rabbit IgG (H+L) secondary antibody, and Alexa Fluor 488 conjugate (A32731) were obtained from Thermo Fisher Scientific. Anti-Myc magnetic beads (B26301) and anti-HA magnetic beads (B26201) were obtained from Bimake. Anti-Flag M2 affinity gel (A2220) was obtained from Sigma-Aldrich. Torin1 (F6101) was obtained from Ubiquitin-Proteasome Biotechnologies.

### Immunoprecipitation and WB.

Cells were harvested and lysed with TAP buffer (20 mM Tris-HCl, pH 7.5, 150 mM NaCl, 0.5% NP-40, 1 mM NaF, 1 mM Na_3_VO_4_, 1 mM EDTA, protease cocktail) for 30 min on ice. The supernatants were collected by centrifugation at 13,000 rpm for 20 min at 4°C. For Flag, Myc, or HA tag IP, tag affinity gel beads were added to the supernatants and incubated overnight. Beads were washed three times with TAP buffer, boiled at 100°C for 10 min in SDS protein loading buffers, and analyzed by Western blotting (WB). Protein concentration was determined based on the Bradford method using the Bio-Rad protein assay kit. Equal amounts of protein were separated by 12% SDS-PAGE and electrophoretically transferred onto a nitrocellulose membrane. After being blocked with 5% nonfat milk in phosphate-buffered saline–Tween 20 (PBST), the membrane was incubated with the primary antibodies, followed by horseradish peroxidase (HRP)-conjugated goat anti-mouse IgG.

### VLP release assay.

To analyze the VLP released from cells, the culture medium of transfected cells was collected and centrifuged at 13,000 rpm for 5 min to remove cell debris, layered onto a cushion of 20% (wt/vol) sucrose in PBS, and subsequently ultracentrifuged on an Optima MAX-XP ultracentrifuge (Beckman) at 35,000 rpm for 2 h at 4°C; the VLPs pelleted at the bottom of the tubes were resuspended in 35 μl of TNE buffer (50 mM Tris-HCl [pH 7.4], 100 mM NaCl, 0.5 mM EDTA [pH 8.0]) overnight at 4°C. Samples were boiled with SDS-PAGE loading buffer and analyzed by WB as described above.

VLP size and particle number were analyzed using a NanoSight NS300 system. The parameters for the process were a screen gain of 10 and a detection threshold of 3.

To measure the VLP size from electron microscopy images, we used correspondence analysis between the scale bar and pixel to calculate the approximate diameters of VLPs.

### Protease protection assay.

VLPs from medium of cells were prepared as described above. Trypsin (GIBCO) was added to a final concentration of 2 μg/ml, along with 1% Triton X-100 if desired. Samples were incubated at 37°C for 1 h and then mixed with SDS-PAGE loading buffer and boiled for WB analysis.

### SARS-CoV-2 virus infection.

All work with live SARS-CoV-2 virus was performed inside biosafety cabinets in the biosafety level 3 facility at Hubei Provincial Center for Disease Control and Prevention. Vero cells in 6-well plates were infected with SARS-CoV-2 (WBP-1) at an MOI of 0.05 PFU/cell for 1 h at 37°C with 5% CO_2_, and then infection medium was removed and replaced with fresh DMEM with 2% FBS.

### Plaque assay.

SARS-CoV-2-containing culture medium was serially 10-fold diluted. Vero cells in 6-well plates were grown to 60% to 70% confluence and infected with 100 μl of the dilutions. Plates were incubated for 2 h at 37°C with 5% CO_2_ and then washed with PBS; the infection medium was replaced with methylcellulose, and plates were incubated at 37°C with 5% CO_2_ for another 3 to 4 days until visible viral plaques were detected. Plates were stained with 0.5% crystal violet for 4 h at room temperature and washed; then the plaques were counted, and the viral titers were calculated.

### Immunofluorescence analysis.

Cells were washed with PBS and fixed with 4% paraformaldehyde for 15 min at room temperature, and then cells were washed three times with PBS and incubated with 0.1% saponin for 10 min. After being washed three times with PBS, cells were blocked with 10% FBS for 30 min. Specific primary antibodies were added and incubated overnight, and cells were then washed with PBS three times, followed by incubation with the goat anti-rabbit IgG rhodamine or goat anti-mouse IgG fluorescein secondary antibody for 1 h. Cells were then washed with PBS three times.

### Transmission electron microscopy.

VLPs were prepared as described above. SARS-CoV-2 was purified via the same protocol with VLP purification, and purified viruses were inactivated by incubating the cultures in the 0.2% methanal overnight. VLPs or native SARS-CoV-2 virions were resuspended in 0.5 ml of TNE buffer, mixed with 4 ml PBS, and then centrifuged at 35,000 rpm for 2 h at 4°C. The final pellets were resuspended in 100 μL of TNE buffer. After centrifugation at 13,000 rpm for 1 min at 4°C to get rid of insoluble materials, samples were absorbed onto a carbon-coated copper grid negatively stained with 1% phosphotungstic acid (pH 7.0) and then analyzed on a transmission electron microscope.

HEK293T cells were transfected with the indicated plasmids for 48 h, or wild-type and RNF5 KD Vero cells were infected with SARS-CoV-2 for 36 h. Cells were then fixed by a fixative liquid (3% [vol/vol] paraformaldehyde, 1.5% [vol/vol] glutaraldehyde, 2.5% [wt/vol] sucrose in 0.1 M sodium phosphate buffer, pH 7.4) for 2 h at room temperature. Then the cells were collected and centrifuged at 4°C. Postfixed with 1% osmium tetroxide for 1 h on ice under dark conditions, cells were incubated with 2% uranyl acetate overnight, dehydrated in increasing concentrations of ethanol (50%, 75%, 95%, and 100%), and processed for embedding in epoxy resin. Ultrathin (70-nm) sections were collected on uncoated 200-mesh copper grids, stained with uranyl acetate and lead citrate, and observed by transmission electron microscopy.

### Quantification and statistical analysis.

Statistical parameters, including the definition and exact values of *n*, distribution, and deviation, are reported in the figure legends. Data are expressed as means ± standard deviations (SD). The significance of the variability between different groups was determined by two-way analyses of variance using GraphPad Prism software. Error bars are means ± SD from two or three independent experiments. With Student’s *t* test, a *P* value of <0.05 was considered statistically significant and a *P* value of >0.05 was considered statistically nonsignificant (NS).

### Data availability.

All data needed to evaluate the conclusions in the paper are present in the paper and/or the supplemental material. Additional data related to this paper may be requested from the corresponding author.
