# Supplementary figures and images for: The E3 Ubiquitin Ligase RNF5 Facilitates SARS-CoV-2 Membrane Protein-Mediated Virion Release
Source: mBio. 2022 Feb 1;13(1):e03168-21. doi: 10.1128/mbio.03168-21 (PMC8805027; doi:10.1128/mbio.03168-21)

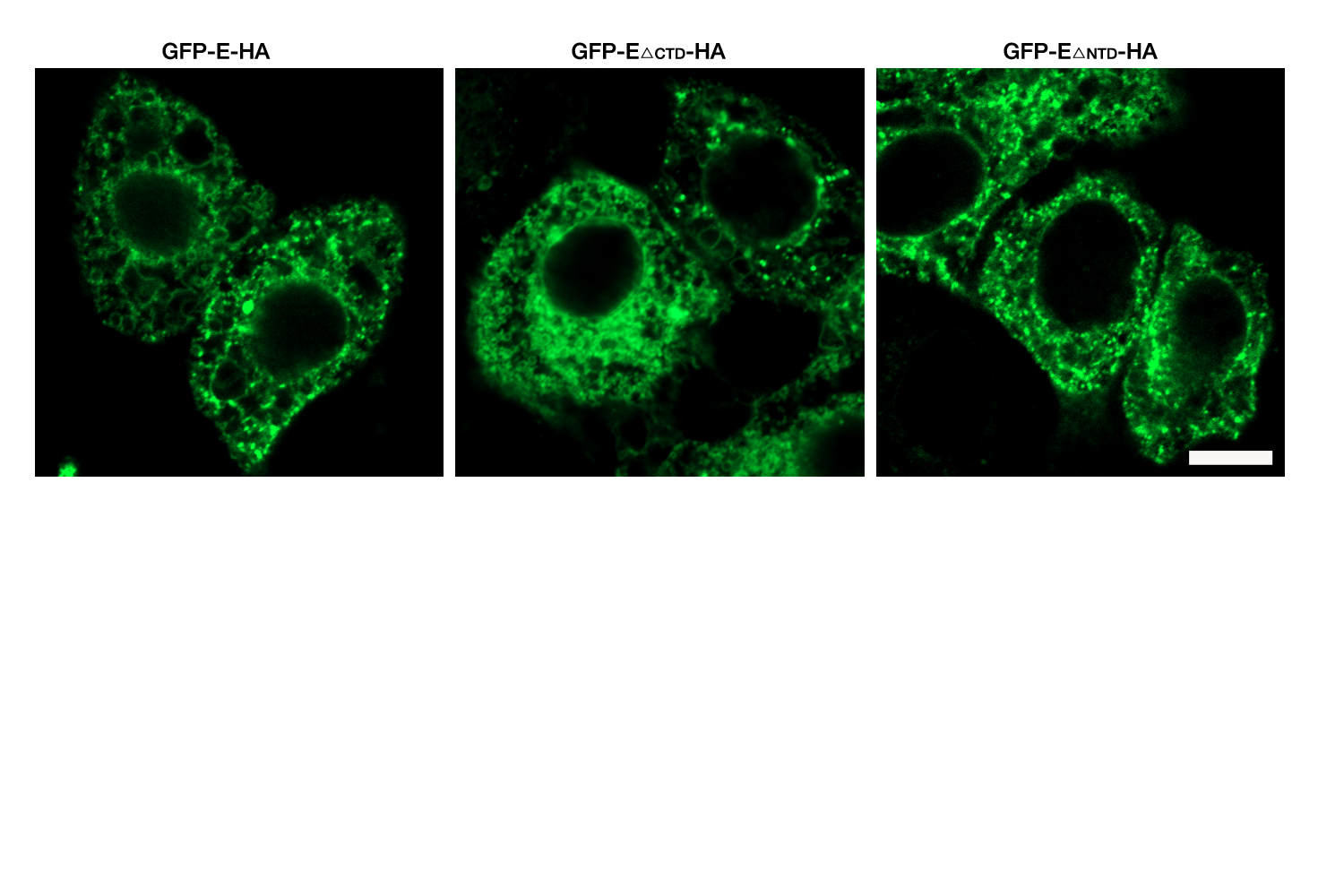

Supplement: FIG S2 [file mbio.03168-21-sf002.tif]

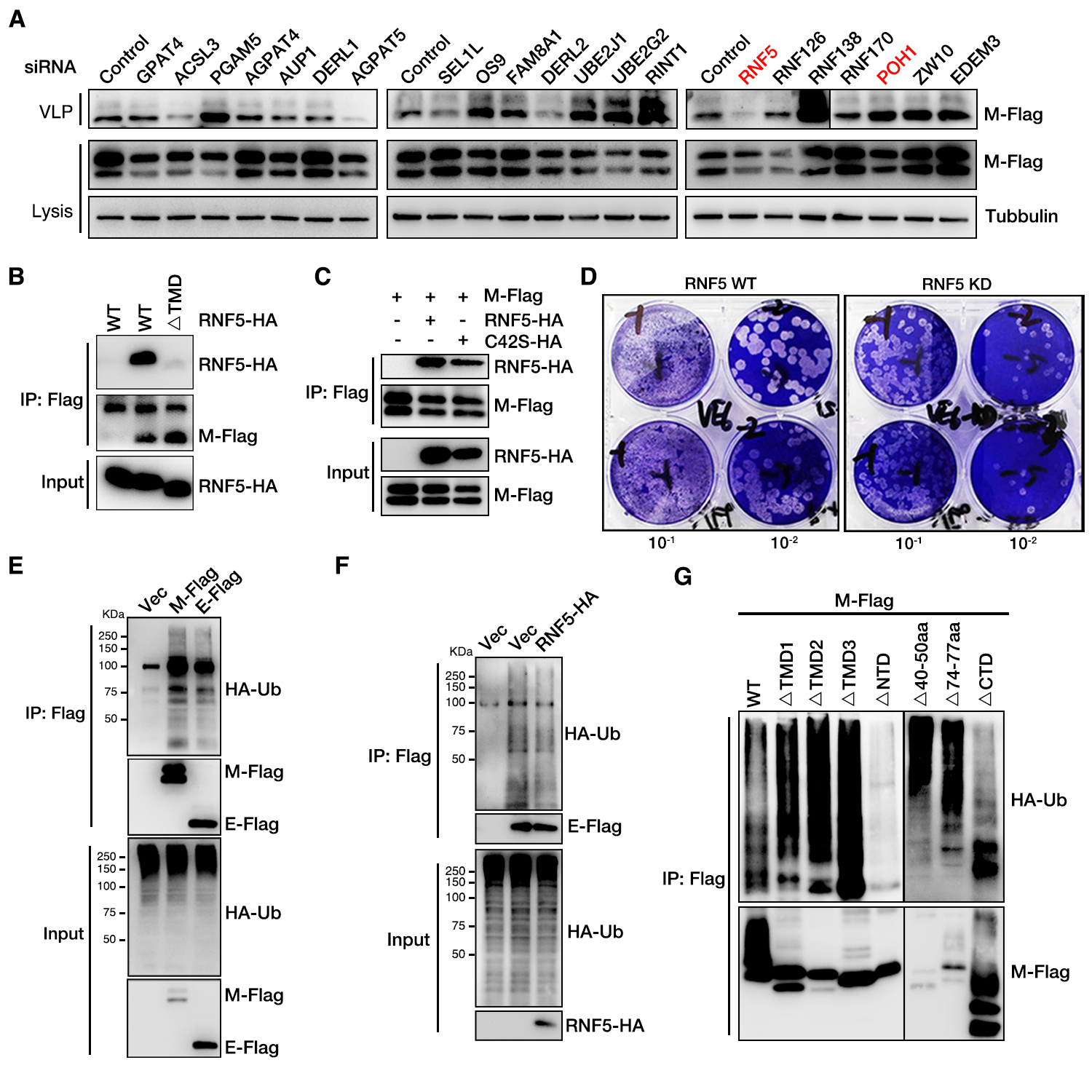

Supplement: FIG S1 [file mbio.03168-21-sf001.tif]
